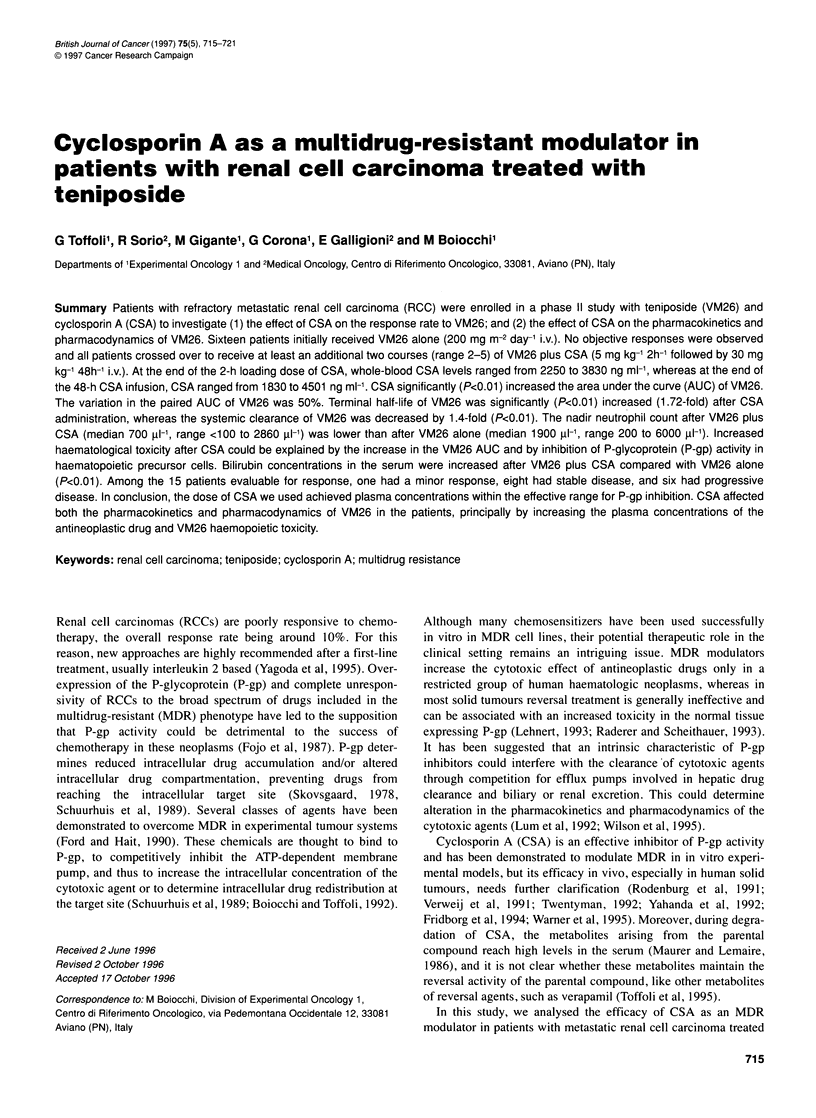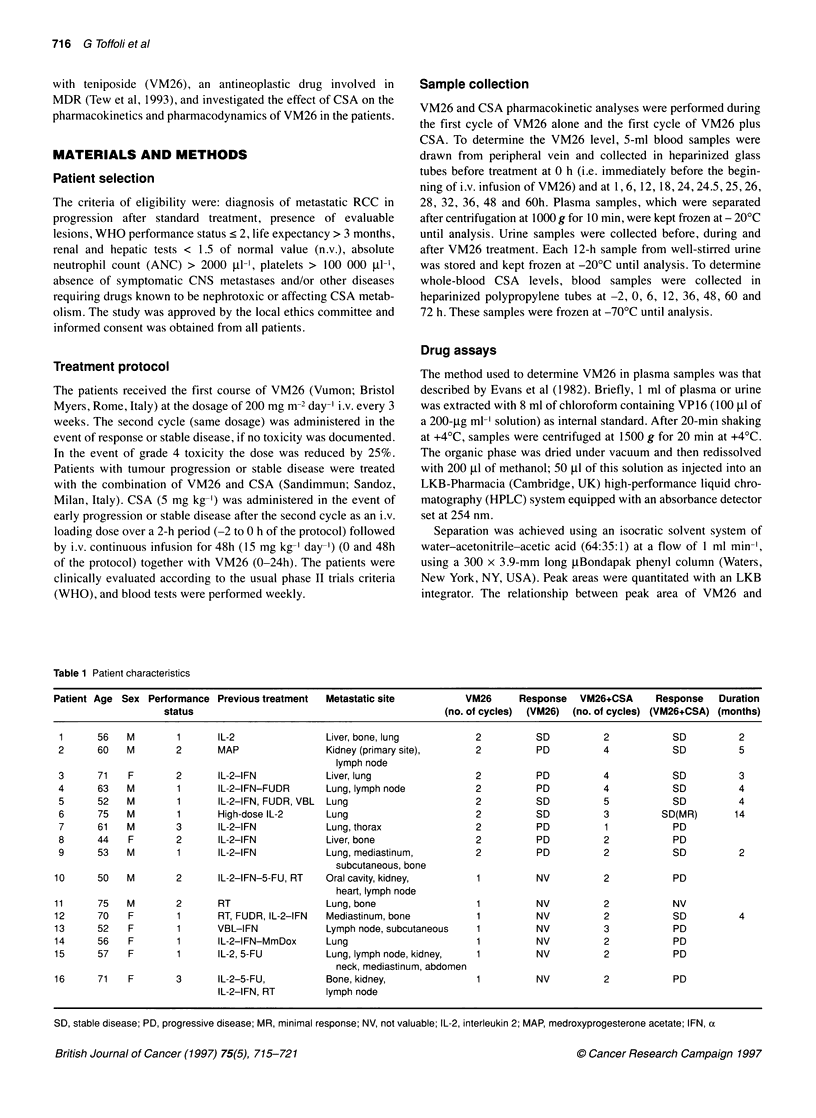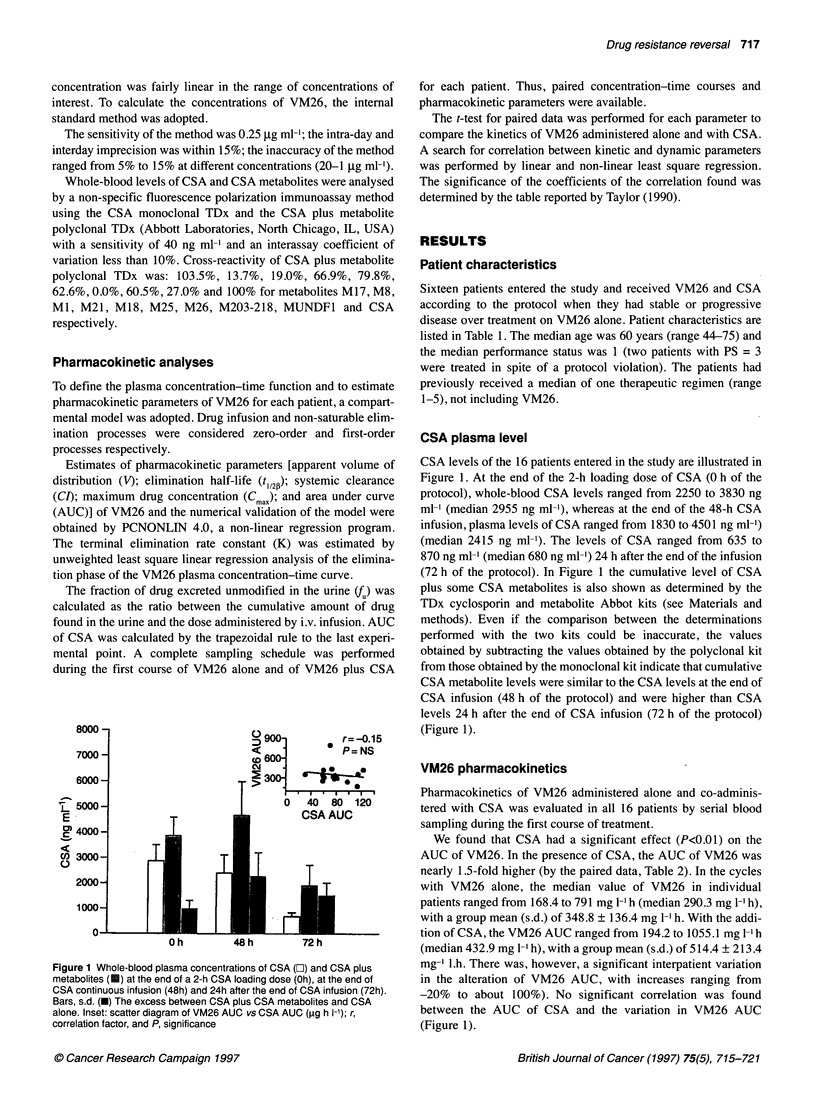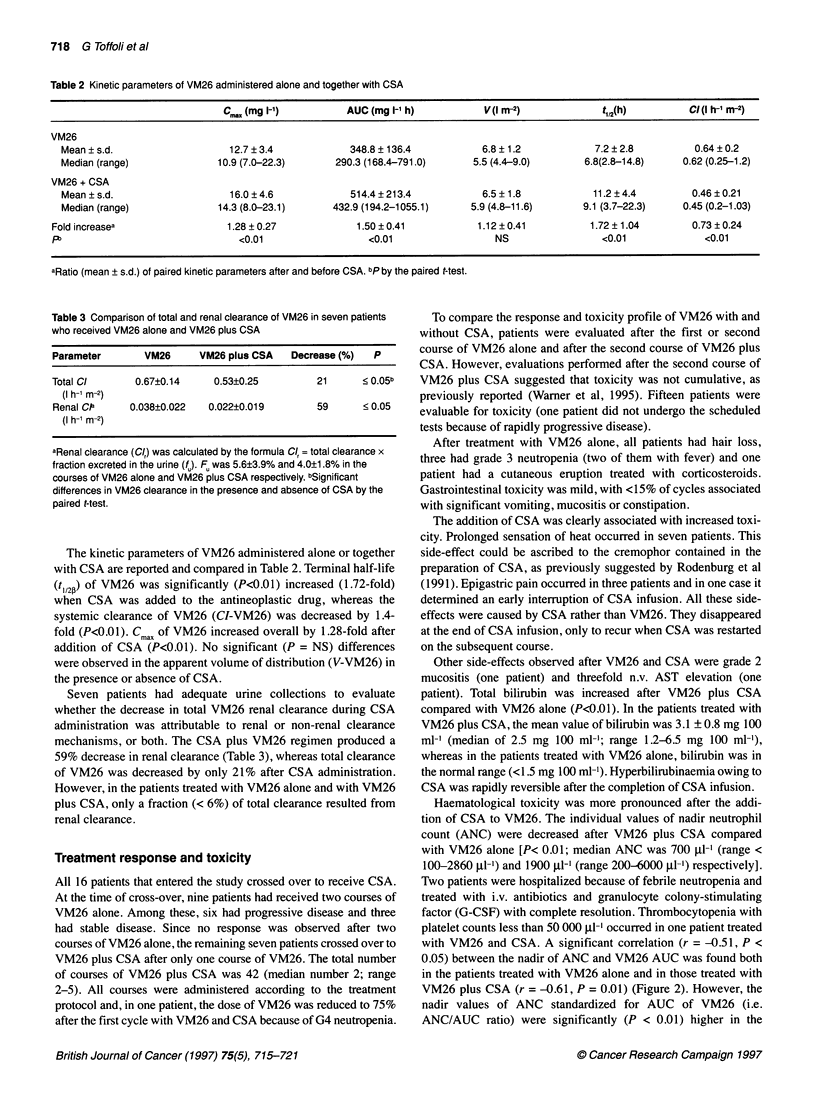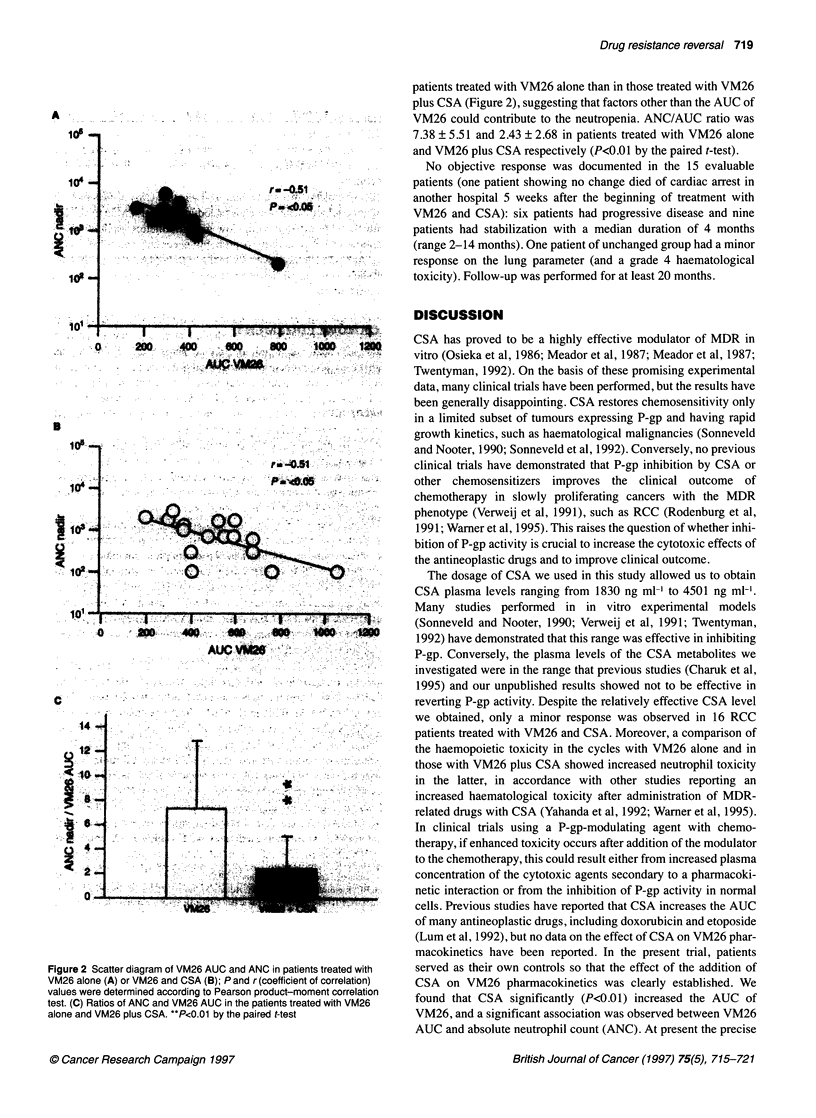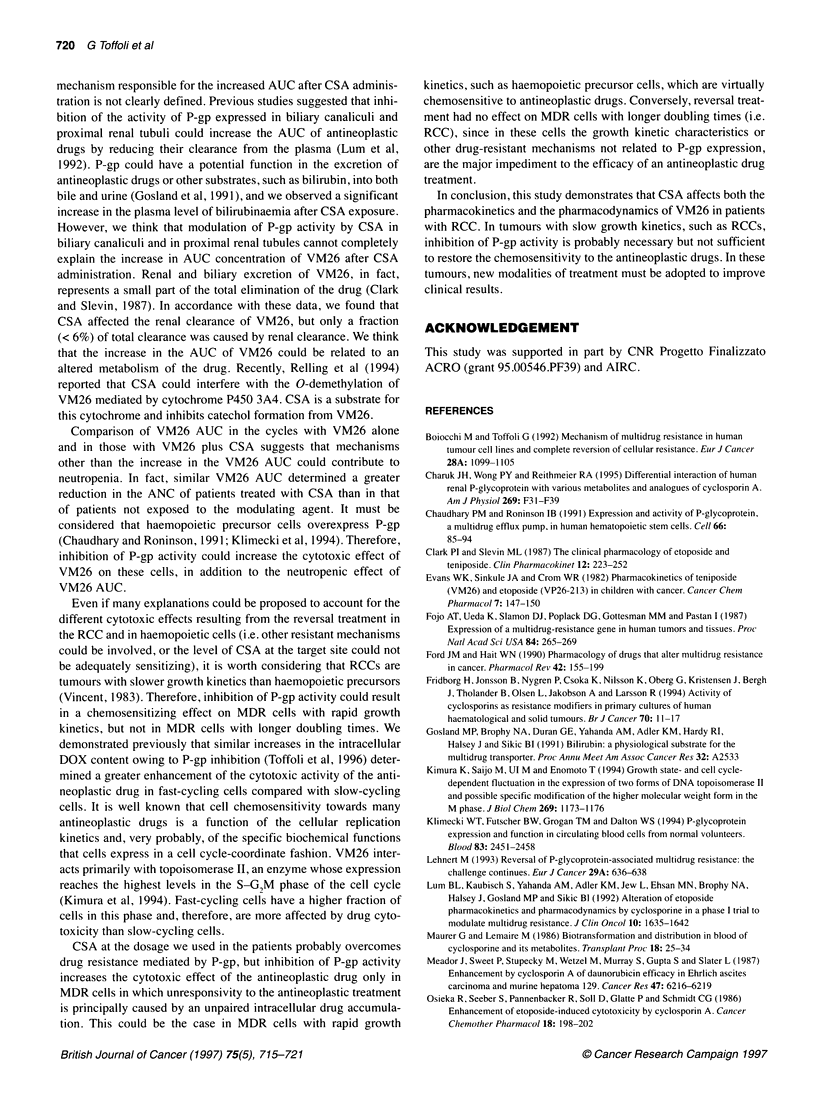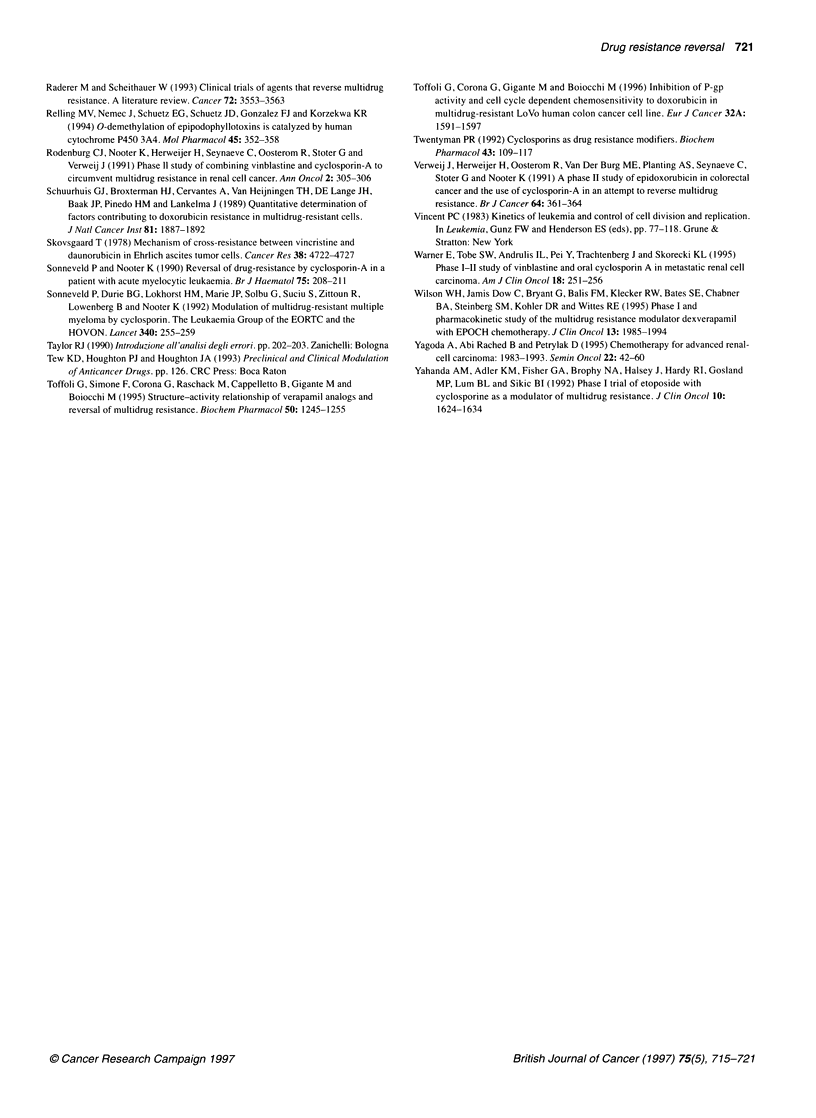# Cyclosporin A as a multidrug-resistant modulator in patients with renal cell carcinoma treated with teniposide.

**DOI:** 10.1038/bjc.1997.127

**Published:** 1997

**Authors:** G. Toffoli, R. Sorio, M. Gigante, G. Corona, E. Galligioni, M. Boiocchi

**Affiliations:** Department of Experimental Oncology, Centro di Riferimento Oncologico, Aviano (PN), Italy.

## Abstract

Patients with refractory metastatic renal cell carcinoma (RCC) were enrolled in a phase II study with teniposide (VM26) and cyclosporin A (CSA) to investigate (1) the effect of CSA on the response rate to VM26; and (2) the effect of CSA on the pharmacokinetics and pharmacodynamics of VM26. Sixteen patients initially received VM26 alone (200 mg m(-2) day(-1) i.v.). No objective responses were observed and all patients crossed over to receive at least an additional two courses (range 2-5) of VM26 plus CSA (5 mg kg(-1) 2h(-1) followed by 30 mg kg(-1) 48h(-1) i.v.). At the end of the 2-h loading dose of CSA, whole-blood CSA levels ranged from 2250 to 3830 ng ml(-1), whereas at the end of the 48-h CSA infusion, CSA ranged from 1830 to 4501 ng ml(-1). CSA significantly (P<0.01) increased the area under the curve (AUC) of VM26. The variation in the paired AUC of VM26 was 50%. Terminal half-life of VM26 was significantly (P<0.01) increased (1.72-fold) after CSA administration, whereas the systemic clearance of VM26 was decreased by 1.4-fold (P<0.01). The nadir neutrophil count after VM26 plus CSA (median 700 microl(-1), range <100 to 2860 microl(-1)) was lower than after VM26 alone (median 1900 microl(-1), range 200 to 6000 microl(-1)). Increased haematological toxicity after CSA could be explained by the increase in the VM26 AUC and by inhibition of P-glycoprotein (P-gp) activity in haematopoietic precursor cells. Bilirubin concentrations in the serum were increased after VM26 plus CSA compared with VM26 alone (P<0.01). Among the 15 patients evaluable for response, one had a minor response, eight had stable disease, and six had progressive disease. In conclusion, the dose of CSA we used achieved plasma concentrations within the effective range for P-gp inhibition. CSA affected both the pharmacokinetics and pharmacodynamics of VM26 in the patients, principally by increasing the plasma concentrations of the antineoplastic drug and VM26 haemopoietic toxicity.


British Journal of Cancer (1997) 75(5), 715–721
© 1997 Cancer Research Campaign

### Cyclosporin A as a multidrug-resistant modulator in patients with renal cell carcinoma treated with teniposide

**G Toffoli[1], R Sorio[2], M Gigante[1], G Corona[1], E Galligioni[2] and M Boiocchi[1]**

Departments of [1]Experimental Oncology 1 and [2]Medical Oncology, Centro di Riferimento Oncologico, 33081, Aviano (PN), Italy

**Summary** Patients with refractory metastatic renal cell carcinoma (RCC) were enrolled in a phase II study with teniposide (VM26) and cyclosporin A (CSA) to investigate (1) the effect of CSA on the response rate to VM26; and (2) the effect of CSA on the pharmacokinetics and pharmacodynamics of VM26. Sixteen patients initially received VM26 alone (200 mg $m^{-2}$ $day^{-1}$ i.v.). No objective responses were observed and all patients crossed over to receive at least an additional two courses (range 2–5) of VM26 plus CSA (5 mg $kg^{-1}$ $2h^{-1}$ followed by 30 mg $kg^{-1}$ $48h^{-1}$ i.v.). At the end of the 2-h loading dose of CSA, whole-blood CSA levels ranged from 2250 to 3830 ng $ml^{-1}$, whereas at the end of the 48-h CSA infusion, CSA ranged from 1830 to 4501 ng $ml^{-1}$. CSA significantly ($P$<0.01) increased the area under the curve (AUC) of VM26. The variation in the paired AUC of VM26 was 50%. Terminal half-life of VM26 was significantly ($P$<0.01) increased (1.72-fold) after CSA administration, whereas the systemic clearance of VM26 was decreased by 1.4-fold ($P$<0.01). The nadir neutrophil count after VM26 plus CSA (median 700 $\mu l^{-1}$, range <100 to 2860 $\mu l^{-1}$) was lower than after VM26 alone (median 1900 $\mu l^{-1}$, range 200 to 6000 $\mu l^{-1}$). Increased haematological toxicity after CSA could be explained by the increase in the VM26 AUC and by inhibition of P-glycoprotein (P-gp) activity in haematopoietic precursor cells. Bilirubin concentrations in the serum were increased after VM26 plus CSA compared with VM26 alone ($P$<0.01). Among the 15 patients evaluable for response, one had a minor response, eight had stable disease, and six had progressive disease. In conclusion, the dose of CSA we used achieved plasma concentrations within the effective range for P-gp inhibition. CSA affected both the pharmacokinetics and pharmacodynamics of VM26 in the patients, principally by increasing the plasma concentrations of the antineoplastic drug and VM26 haemopoietic toxicity.

**Keywords:** renal cell carcinoma; teniposide; cyclosporin A; multidrug resistance

Renal cell carcinomas (RCCs) are poorly responsive to chemotherapy, the overall response rate being around 10%. For this reason, new approaches are highly recommended after a first-line treatment, usually interleukin 2 based (Yagoda et al, 1995). Overexpression of the P-glycoprotein (P-gp) and complete unresponsivity of RCCs to the broad spectrum of drugs included in the multidrug-resistant (MDR) phenotype have led to the supposition that P-gp activity could be detrimental to the success of chemotherapy in these neoplasms (Fojo et al, 1987). P-gp determines reduced intracellular drug accumulation and/or altered intracellular drug compartmentation, preventing drugs from reaching the intracellular target site (Skovsgaard, 1978, Schuurhuis et al, 1989). Several classes of agents have been demonstrated to overcome MDR in experimental tumour systems (Ford and Hait, 1990). These chemicals are thought to bind to P-gp, to competitively inhibit the ATP-dependent membrane pump, and thus to increase the intracellular concentration of the cytotoxic agent or to determine intracellular drug redistribution at the target site (Schuurhuis et al, 1989; Boiocchi and Toffoli, 1992).

*Received 2 June 1996*
*Revised 2 October 1996*
*Accepted 17 October 1996*

*Correspondence to:* M Boiocchi, Division of Experimental Oncology 1, Centro di Riferimento Oncologico, via Pedemontana Occidentale 12, 33081 Aviano (PN), Italy

Although many chemosensitizers have been used successfully in vitro in MDR cell lines, their potential therapeutic role in the clinical setting remains an intriguing issue. MDR modulators increase the cytotoxic effect of antineoplastic drugs only in a restricted group of human haematologic neoplasms, whereas in most solid tumours reversal treatment is generally ineffective and can be associated with an increased toxicity in the normal tissue expressing P-gp (Lehnert, 1993; Raderer and Scheithauer, 1993). It has been suggested that an intrinsic characteristic of P-gp inhibitors could interfere with the clearance of cytotoxic agents through competition for efflux pumps involved in hepatic drug clearance and biliary or renal excretion. This could determine alteration in the pharmacokinetics and pharmacodynamics of the cytotoxic agents (Lum et al, 1992; Wilson et al, 1995).

Cyclosporin A (CSA) is an effective inhibitor of P-gp activity and has been demonstrated to modulate MDR in in vitro experimental models, but its efficacy in vivo, especially in human solid tumours, needs further clarification (Rodenburg et al, 1991; Verweij et al, 1991; Twentyman, 1992; Yahanda et al, 1992; Fridborg et al, 1994; Warner et al, 1995). Moreover, during degradation of CSA, the metabolites arising from the parental compound reach high levels in the serum (Maurer and Lemaire, 1986), and it is not clear whether these metabolites maintain the reversal activity of the parental compound, like other metabolites of reversal agents, such as verapamil (Toffoli et al, 1995).

In this study, we analysed the efficacy of CSA as an MDR modulator in patients with metastatic renal cell carcinoma treated with teniposide (VM26), an antineoplastic drug involved in MDR (Tew et al, 1993), and investigated the effect of CSA on the pharmacokinetics and pharmacodynamics of VM26 in the patients.

### MATERIALS AND METHODS

### Patient selection

The criteria of eligibility were: diagnosis of metastatic RCC in progression after standard treatment, presence of evaluable lesions, WHO performance status ≤ 2, life expectancy > 3 months, renal and hepatic tests < 1.5 of normal value (n.v.), absolute neutrophil count (ANC) > 2000 $\mu l^{-1}$, platelets > 100 000 $\mu l^{-1}$, absence of symptomatic CNS metastases and/or other diseases requiring drugs known to be nephrotoxic or affecting CSA metabolism. The study was approved by the local ethics committee and informed consent was obtained from all patients.

### Treatment protocol

The patients received the first course of VM26 (Vumon; Bristol Myers, Rome, Italy) at the dosage of 200 mg $m^{-2}$ $day^{-1}$ i.v. every 3 weeks. The second cycle (same dosage) was administered in the event of response or stable disease, if no toxicity was documented. In the event of grade 4 toxicity the dose was reduced by 25%. Patients with tumour progression or stable disease were treated with the combination of VM26 and CSA (Sandimmun; Sandoz, Milan, Italy). CSA (5 mg $kg^{-1}$) was administered in the event of early progression or stable disease after the second cycle as an i.v. loading dose over a 2-h period (–2 to 0 h of the protocol) followed by i.v. continuous infusion for 48h (15 mg $kg^{-1}$ $day^{-1}$) (0 and 48h of the protocol) together with VM26 (0–24h). The patients were clinically evaluated according to the usual phase II trials criteria (WHO), and blood tests were performed weekly.

### Sample collection

VM26 and CSA pharmacokinetic analyses were performed during the first cycle of VM26 alone and the first cycle of VM26 plus CSA. To determine the VM26 level, 5-ml blood samples were drawn from peripheral vein and collected in heparinized glass tubes before treatment at 0 h (i.e. immediately before the beginning of i.v. infusion of VM26) and at 1, 6, 12, 18, 24, 24.5, 25, 26, 28, 32, 36, 48 and 60h. Plasma samples, which were separated after centrifugation at 1000 ***g*** for 10 min, were kept frozen at – 20°C until analysis. Urine samples were collected before, during and after VM26 treatment. Each 12-h sample from well-stirred urine was stored and kept frozen at –20°C until analysis. To determine whole-blood CSA levels, blood samples were collected in heparinized polypropylene tubes at –2, 0, 6, 12, 36, 48, 60 and 72 h. These samples were frozen at –70°C until analysis.

### Drug assays

The method used to determine VM26 in plasma samples was that described by Evans et al (1982). Briefly, 1 ml of plasma or urine was extracted with 8 ml of chloroform containing VP16 (100 µl of a 200-µg $ml^{-1}$ solution) as internal standard. After 20-min shaking at +4°C, samples were centrifuged at 1500 ***g*** for 20 min at +4°C. The organic phase was dried under vacuum and then redissolved with 200 µl of methanol; 50 µl of this solution as injected into an LKB-Pharmacia (Cambridge, UK) high-performance liquid chromatography (HPLC) system equipped with an absorbance detector set at 254 nm.

Separation was achieved using an isocratic solvent system of water–acetonitrile–acetic acid (64:35:1) at a flow of 1 ml $min^{-1}$, using a 300 × 3.9-mm long µBondapak phenyl column (Waters, New York, NY, USA). Peak areas were quantitated with an LKB integrator. The relationship between peak area of VM26 and

**Table 1** Patient characteristics

| Patient | Age | Sex | Performance status | Previous treatment | Metastatic site | VM26 (no. of cycles) | Response (VM26) | VM26+CSA (no. of cycles) | Response (VM26+CSA) | Duration (months) |
|---|---|---|---|---|---|---|---|---|---|---|
| 1 | 56 | M | 1 | IL-2 | Liver, bone, lung | 2 | SD | 2 | SD | 2 |
| 2 | 60 | M | 2 | MAP | Kidney (primary site), lymph node | 2 | PD | 4 | SD | 5 |
| 3 | 71 | F | 2 | IL-2–IFN | Liver, lung | 2 | PD | 4 | SD | 3 |
| 4 | 63 | M | 1 | IL-2–IFN–FUDR | Lung, lymph node | 2 | PD | 4 | SD | 4 |
| 5 | 52 | M | 1 | IL-2–IFN, FUDR, VBL | Lung | 2 | SD | 5 | SD | 4 |
| 6 | 75 | M | 1 | High-dose IL-2 | Lung | 2 | SD | 3 | SD(MR) | 14 |
| 7 | 61 | M | 3 | IL-2–IFN | Lung, thorax | 2 | PD | 1 | PD | |
| 8 | 44 | F | 2 | IL-2–IFN | Liver, bone | 2 | PD | 2 | PD | |
| 9 | 53 | M | 1 | IL-2–IFN | Lung, mediastinum, subcutaneous, bone | 2 | PD | 2 | SD | 2 |
| 10 | 50 | M | 2 | IL-2–IFN–5-FU, RT | Oral cavity, kidney, heart, lymph node | 1 | NV | 2 | PD | |
| 11 | 75 | M | 2 | RT | Lung, bone | 1 | NV | 2 | NV | |
| 12 | 70 | F | 1 | RT, FUDR, IL-2–IFN | Mediastinum, bone | 1 | NV | 2 | SD | 4 |
| 13 | 52 | F | 1 | VBL–IFN | Lymph node, subcutaneous | 1 | NV | 3 | PD | |
| 14 | 56 | F | 1 | IL-2–IFN–MmDox | Lung | 1 | NV | 2 | PD | |
| 15 | 57 | F | 1 | IL-2, 5-FU | Lung, lymph node, kidney, neck, mediastinum, abdomen | 1 | NV | 2 | PD | |
| 16 | 71 | F | 3 | IL-2–5-FU, IL-2–IFN, RT | Bone, kidney, lymph node | 1 | NV | 2 | PD | |

SD, stable disease; PD, progressive disease; MR, minimal response; NV, not valuable; IL-2, interleukin 2; MAP, medroxyprogesterone acetate; IFN, α

 © Cancer Research Campaign 1997

concentration was fairly linear in the range of concentrations of interest. To calculate the concentrations of VM26, the internal standard method was adopted.

The sensitivity of the method was 0.25 μg ml$^{-1}$; the intra-day and interday imprecision was within 15%; the inaccuracy of the method ranged from 5% to 15% at different concentrations (20–1 μg ml$^{-1}$).

Whole-blood levels of CSA and CSA metabolites were analysed by a non-specific fluorescence polarization immunoassay method using the CSA monoclonal TDx and the CSA plus metabolite polyclonal TDx (Abbott Laboratories, North Chicago, IL, USA) with a sensitivity of 40 ng ml$^{-1}$ and an interassay coefficient of variation less than 10%. Cross-reactivity of CSA plus metabolite polyclonal TDx was: 103.5%, 13.7%, 19.0%, 66.9%, 79.8%, 62.6%, 0.0%, 60.5%, 27.0% and 100% for metabolites M17, M8, M1, M21, M18, M25, M26, M203-218, MUNDF1 and CSA respectively.

### Pharmacokinetic analyses

To define the plasma concentration–time function and to estimate pharmacokinetic parameters of VM26 for each patient, a compartmental model was adopted. Drug infusion and non-saturable elimination processes were considered zero-order and first-order processes respectively.

Estimates of pharmacokinetic parameters [apparent volume of distribution ($V$); elimination half-life ($t_{1/2\beta}$); systemic clearance ($Cl$); maximum drug concentration ($C_{max}$); and area under curve (AUC)] of VM26 and the numerical validation of the model were obtained by PCNONLIN 4.0, a non-linear regression program. The terminal elimination rate constant (K) was estimated by unweighted least square linear regression analysis of the elimination phase of the VM26 plasma concentration–time curve.

The fraction of drug excreted unmodified in the urine ($f_u$) was calculated as the ratio between the cumulative amount of drug found in the urine and the dose administered by i.v. infusion. AUC of CSA was calculated by the trapezoidal rule to the last experimental point. A complete sampling schedule was performed during the first course of VM26 alone and of VM26 plus CSA for each patient. Thus, paired concentration–time courses and pharmacokinetic parameters were available.

The $t$-test for paired data was performed for each parameter to compare the kinetics of VM26 administered alone and with CSA. A search for correlation between kinetic and dynamic parameters was performed by linear and non-linear least square regression. The significance of the coefficients of the correlation found was determined by the table reported by Taylor (1990).

**Figure 1** Whole-blood plasma concentrations of CSA (□) and CSA plus metabolites (▨) at the end of a 2-h CSA loading dose (0h), at the end of CSA continuous infusion (48h) and 24h after the end of CSA infusion (72h). Bars, s.d. (■) The excess between CSA plus CSA metabolites and CSA alone. Inset: scatter diagram of VM26 AUC *vs* CSA AUC (μg h l$^{-1}$); $r$, correlation factor, and $P$, significance

### RESULTS

### Patient characteristics

Sixteen patients entered the study and received VM26 and CSA according to the protocol when they had stable or progressive disease over treatment on VM26 alone. Patient characteristics are listed in Table 1. The median age was 60 years (range 44–75) and the median performance status was 1 (two patients with PS = 3 were treated in spite of a protocol violation). The patients had previously received a median of one therapeutic regimen (range 1–5), not including VM26.

### CSA plasma level

CSA levels of the 16 patients entered in the study are illustrated in Figure 1. At the end of the 2-h loading dose of CSA (0 h of the protocol), whole-blood CSA levels ranged from 2250 to 3830 ng ml$^{-1}$ (median 2955 ng ml$^{-1}$), whereas at the end of the 48-h CSA infusion, plasma levels of CSA ranged from 1830 to 4501 ng ml$^{-1}$) (median 2415 ng ml$^{-1}$). The levels of CSA ranged from 635 to 870 ng ml$^{-1}$ (median 680 ng ml$^{-1}$) 24 h after the end of the infusion (72 h of the protocol). In Figure 1 the cumulative level of CSA plus some CSA metabolites is also shown as determined by the TDx cyclosporin and metabolite Abbot kits (see Materials and methods). Even if the comparison between the determinations performed with the two kits could be inaccurate, the values obtained by subtracting the values obtained by the polyclonal kit from those obtained by the monoclonal kit indicate that cumulative CSA metabolite levels were similar to the CSA levels at the end of CSA infusion (48 h of the protocol) and were higher than CSA levels 24 h after the end of CSA infusion (72 h of the protocol) (Figure 1).

### VM26 pharmacokinetics

Pharmacokinetics of VM26 administered alone and co-administered with CSA was evaluated in all 16 patients by serial blood sampling during the first course of treatment.

We found that CSA had a significant effect ($P$<0.01) on the AUC of VM26. In the presence of CSA, the AUC of VM26 was nearly 1.5-fold higher (by the paired data, Table 2). In the cycles with VM26 alone, the median value of VM26 in individual patients ranged from 168.4 to 791 mg l$^{-1}$ h (median 290.3 mg l$^{-1}$ h), with a group mean (s.d.) of 348.8 ± 136.4 mg l$^{-1}$ h. With the addition of CSA, the VM26 AUC ranged from 194.2 to 1055.1 mg l$^{-1}$ h (median 432.9 mg l$^{-1}$ h), with a group mean (s.d.) of 514.4 ± 213.4 mg$^{-1}$ l.h. There was, however, a significant interpatient variation in the alteration of VM26 AUC, with increases ranging from –20% to about 100%). No significant correlation was found between the AUC of CSA and the variation in VM26 AUC (Figure 1).

© Cancer Research Campaign 1997

**Table 2** Kinetic parameters of VM26 administered alone and together with CSA

| | $C_{max}$ (mg l$^{-1}$) | AUC (mg l$^{-1}$ h) | $V$ (l m$^{-2}$) | $t_{1/2}$(h) | $Cl$ (l h$^{-1}$ m$^{-2}$) |
|---|---|---|---|---|---|
| VM26 | | | | | |
| Mean ± s.d. | 12.7 ± 3.4 | 348.8 ± 136.4 | 6.8 ± 1.2 | 7.2 ± 2.8 | 0.64 ± 0.2 |
| Median (range) | 10.9 (7.0–22.3) | 290.3 (168.4–791.0) | 5.5 (4.4–9.0) | 6.8(2.8–14.8) | 0.62 (0.25–1.2) |
| VM26 + CSA | | | | | |
| Mean ± s.d. | 16.0 ± 4.6 | 514.4 ± 213.4 | 6.5 ± 1.8 | 11.2 ± 4.4 | 0.46 ± 0.21 |
| Median (range) | 14.3 (8.0–23.1) | 432.9 (194.2–1055.1) | 5.9 (4.8–11.6) | 9.1 (3.7–22.3) | 0.45 (0.2–1.03) |
| Fold increase[a] | 1.28 ± 0.27 | 1.50 ± 0.41 | 1.12 ± 0.41 | 1.72 ± 1.04 | 0.73 ± 0.24 |
| $P$[b] | <0.01 | <0.01 | NS | <0.01 | <0.01 |

[a]Ratio (mean ± s.d.) of paired kinetic parameters after and before CSA. [b]$P$ by the paired $t$-test.

**Table 3** Comparison of total and renal clearance of VM26 in seven patients who received VM26 alone and VM26 plus CSA

| Parameter | VM26 | VM26 plus CSA | Decrease (%) | $P$ |
|---|---|---|---|---|
| Total $Cl$ (l h$^{-1}$ m$^{-2}$) | 0.67±0.14 | 0.53±0.25 | 21 | ≤ 0.05[b] |
| Renal $Cl$[a] (l h$^{-1}$ m$^{-2}$) | 0.038±0.022 | 0.022±0.019 | 59 | ≤ 0.05 |

[a]Renal clearance ($Cl_r$) was calculated by the formula $Cl_r$ = total clearance × fraction excreted in the urine ($f_u$). $F_u$ was 5.6±3.9% and 4.0±1.8% in the courses of VM26 alone and VM26 plus CSA respectively. [b]Significant differences in VM26 clearance in the presence and absence of CSA by the paired $t$-test.

The kinetic parameters of VM26 administered alone or together with CSA are reported and compared in Table 2. Terminal half-life ($t_{1/2\beta}$) of VM26 was significantly ($P$<0.01) increased (1.72-fold) when CSA was added to the antineoplastic drug, whereas the systemic clearance of VM26 ($Cl$-VM26) was decreased by 1.4-fold ($P$<0.01). $C_{max}$ of VM26 increased overall by 1.28-fold after addition of CSA ($P$<0.01). No significant ($P$ = NS) differences were observed in the apparent volume of distribution ($V$-VM26) in the presence or absence of CSA.

Seven patients had adequate urine collections to evaluate whether the decrease in total VM26 renal clearance during CSA administration was attributable to renal or non-renal clearance mechanisms, or both. The CSA plus VM26 regimen produced a 59% decrease in renal clearance (Table 3), whereas total clearance of VM26 was decreased by only 21% after CSA administration. However, in the patients treated with VM26 alone and with VM26 plus CSA, only a fraction (< 6%) of total clearance resulted from renal clearance.

### Treatment response and toxicity

All 16 patients that entered the study crossed over to receive CSA. At the time of cross-over, nine patients had received two courses of VM26 alone. Among these, six had progressive disease and three had stable disease. Since no response was observed after two courses of VM26 alone, the remaining seven patients crossed over to VM26 plus CSA after only one course of VM26. The total number of courses of VM26 plus CSA was 42 (median number 2; range 2–5). All courses were administered according to the treatment protocol and, in one patient, the dose of VM26 was reduced to 75% after the first cycle with VM26 and CSA because of G4 neutropenia.

To compare the response and toxicity profile of VM26 with and without CSA, patients were evaluated after the first or second course of VM26 alone and after the second course of VM26 plus CSA. However, evaluations performed after the second course of VM26 plus CSA suggested that toxicity was not cumulative, as previously reported (Warner et al, 1995). Fifteen patients were evaluable for toxicity (one patient did not undergo the scheduled tests because of rapidly progressive disease).

After treatment with VM26 alone, all patients had hair loss, three had grade 3 neutropenia (two of them with fever) and one patient had a cutaneous eruption treated with corticosteroids. Gastrointestinal toxicity was mild, with <15% of cycles associated with significant vomiting, mucositis or constipation.

The addition of CSA was clearly associated with increased toxicity. Prolonged sensation of heat occurred in seven patients. This side-effect could be ascribed to the cremophor contained in the preparation of CSA, as previously suggested by Rodenburg et al (1991). Epigastric pain occurred in three patients and in one case it determined an early interruption of CSA infusion. All these side-effects were caused by CSA rather than VM26. They disappeared at the end of CSA infusion, only to recur when CSA was restarted on the subsequent course.

Other side-effects observed after VM26 and CSA were grade 2 mucositis (one patient) and threefold n.v. AST elevation (one patient). Total bilirubin was increased after VM26 plus CSA compared with VM26 alone ($P$<0.01). In the patients treated with VM26 plus CSA, the mean value of bilirubin was 3.1 ± 0.8 mg 100 ml$^{-1}$ (median of 2.5 mg 100 ml$^{-1}$; range 1.2–6.5 mg 100 ml$^{-1}$), whereas in the patients treated with VM26 alone, bilirubin was in the normal range (<1.5 mg 100 ml$^{-1}$). Hyperbilirubinaemia owing to CSA was rapidly reversible after the completion of CSA infusion.

Haematological toxicity was more pronounced after the addition of CSA to VM26. The individual values of nadir neutrophil count (ANC) were decreased after VM26 plus CSA compared with VM26 alone [$P$< 0.01; median ANC was 700 μl$^{-1}$ (range < 100–2860 μl$^{-1}$) and 1900 μl$^{-1}$ (range 200–6000 μl$^{-1}$) respectively]. Two patients were hospitalized because of febrile neutropenia and treated with i.v. antibiotics and granulocyte colony-stimulating factor (G-CSF) with complete resolution. Thrombocytopenia with platelet counts less than 50 000 μl$^{-1}$ occurred in one patient treated with VM26 and CSA. A significant correlation ($r$ = –0.51, $P$ < 0.05) between the nadir of ANC and VM26 AUC was found both in the patients treated with VM26 alone and in those treated with VM26 plus CSA ($r$ = –0.61, $P$ = 0.01) (Figure 2). However, the nadir values of ANC standardized for AUC of VM26 (i.e. ANC/AUC ratio) were significantly ($P$ < 0.01) higher in the

© Cancer Research Campaign 1997

**Figure 2** Scatter diagram of VM26 AUC and ANC in patients treated with VM26 alone (**A**) or VM26 and CSA (**B**); *P* and *r* (coefficient of correlation) values were determined according to Pearson product–moment correlation test. (**C**) Ratios of ANC and VM26 AUC in the patients treated with VM26 alone and VM26 plus CSA. $^{**}P<0.01$ by the paired *t*-test patients treated with VM26 alone than in those treated with VM26 plus CSA (Figure 2), suggesting that factors other than the AUC of VM26 could contribute to the neutropenia. ANC/AUC ratio was $7.38 \pm 5.51$ and $2.43 \pm 2.68$ in patients treated with VM26 alone and VM26 plus CSA respectively ($P<0.01$ by the paired *t*-test).

No objective response was documented in the 15 evaluable patients (one patient showing no change died of cardiac arrest in another hospital 5 weeks after the beginning of treatment with VM26 and CSA): six patients had progressive disease and nine patients had stabilization with a median duration of 4 months (range 2–14 months). One patient of unchanged group had a minor response on the lung parameter (and a grade 4 haematological toxicity). Follow-up was performed for at least 20 months.

### DISCUSSION

CSA has proved to be a highly effective modulator of MDR in vitro (Osieka et al, 1986; Meador et al, 1987; Meador et al, 1987; Twentyman, 1992). On the basis of these promising experimental data, many clinical trials have been performed, but the results have been generally disappointing. CSA restores chemosensitivity only in a limited subset of tumours expressing P-gp and having rapid growth kinetics, such as haematological malignancies (Sonneveld and Nooter, 1990; Sonneveld et al, 1992). Conversely, no previous clinical trials have demonstrated that P-gp inhibition by CSA or other chemosensitizers improves the clinical outcome of chemotherapy in slowly proliferating cancers with the MDR phenotype (Verweij et al, 1991), such as RCC (Rodenburg et al, 1991; Warner et al, 1995). This raises the question of whether inhibition of P-gp activity is crucial to increase the cytotoxic effects of the antineoplastic drugs and to improve clinical outcome.

The dosage of CSA we used in this study allowed us to obtain CSA plasma levels ranging from 1830 ng $ml^{-1}$ to 4501 ng $ml^{-1}$. Many studies performed in in vitro experimental models (Sonneveld and Nooter, 1990; Verweij et al, 1991; Twentyman, 1992) have demonstrated that this range was effective in inhibiting P-gp. Conversely, the plasma levels of the CSA metabolites we investigated were in the range that previous studies (Charuk et al, 1995) and our unpublished results showed not to be effective in reverting P-gp activity. Despite the relatively effective CSA level we obtained, only a minor response was observed in 16 RCC patients treated with VM26 and CSA. Moreover, a comparison of the haemopoietic toxicity in the cycles with VM26 alone and in those with VM26 plus CSA showed increased neutrophil toxicity in the latter, in accordance with other studies reporting an increased haematological toxicity after administration of MDR-related drugs with CSA (Yahanda et al, 1992; Warner et al, 1995). In clinical trials using a P-gp-modulating agent with chemotherapy, if enhanced toxicity occurs after addition of the modulator to the chemotherapy, this could result either from increased plasma concentration of the cytotoxic agents secondary to a pharmacokinetic interaction or from the inhibition of P-gp activity in normal cells. Previous studies have reported that CSA increases the AUC of many antineoplastic drugs, including doxorubicin and etoposide (Lum et al, 1992), but no data on the effect of CSA on VM26 pharmacokinetics have been reported. In the present trial, patients served as their own controls so that the effect of the addition of CSA on VM26 pharmacokinetics was clearly established. We found that CSA significantly ($P<0.01$) increased the AUC of VM26, and a significant association was observed between VM26 AUC and absolute neutrophil count (ANC). At present the precise

© Cancer Research Campaign 1997

mechanism responsible for the increased AUC after CSA administration is not clearly defined. Previous studies suggested that inhibition of the activity of P-gp expressed in biliary canaliculi and proximal renal tubuli could increase the AUC of antineoplastic drugs by reducing their clearance from the plasma (Lum et al, 1992). P-gp could have a potential function in the excretion of antineoplastic drugs or other substrates, such as bilirubin, into both bile and urine (Gosland et al, 1991), and we observed a significant increase in the plasma level of bilirubinaemia after CSA exposure. However, we think that modulation of P-gp activity by CSA in biliary canaliculi and in proximal renal tubules cannot completely explain the increase in AUC concentration of VM26 after CSA administration. Renal and biliary excretion of VM26, in fact, represents a small part of the total elimination of the drug (Clark and Slevin, 1987). In accordance with these data, we found that CSA affected the renal clearance of VM26, but only a fraction (< 6%) of total clearance was caused by renal clearance. We think that the increase in the AUC of VM26 could be related to an altered metabolism of the drug. Recently, Relling et al (1994) reported that CSA could interfere with the *O*-demethylation of VM26 mediated by cytochrome P450 3A4. CSA is a substrate for this cytochrome and inhibits catechol formation from VM26.

Comparison of VM26 AUC in the cycles with VM26 alone and in those with VM26 plus CSA suggests that mechanisms other than the increase in the VM26 AUC could contribute to neutropenia. In fact, similar VM26 AUC determined a greater reduction in the ANC of patients treated with CSA than in that of patients not exposed to the modulating agent. It must be considered that haemopoietic precursor cells overexpress P-gp (Chaudhary and Roninson, 1991; Klimecki et al, 1994). Therefore, inhibition of P-gp activity could increase the cytotoxic effect of VM26 on these cells, in addition to the neutropenic effect of VM26 AUC.

Even if many explanations could be proposed to account for the different cytotoxic effects resulting from the reversal treatment in the RCC and in haemopoietic cells (i.e. other resistant mechanisms could be involved, or the level of CSA at the target site could not be adequately sensitizing), it is worth considering that RCCs are tumours with slower growth kinetics than haemopoietic precursors (Vincent, 1983). Therefore, inhibition of P-gp activity could result in a chemosensitizing effect on MDR cells with rapid growth kinetics, but not in MDR cells with longer doubling times. We demonstrated previously that similar increases in the intracellular DOX content owing to P-gp inhibition (Toffoli et al, 1996) determined a greater enhancement of the cytotoxic activity of the antineoplastic drug in fast-cycling cells compared with slow-cycling cells. It is well known that cell chemosensitivity towards many antineoplastic drugs is a function of the cellular replication kinetics and, very probably, of the specific biochemical functions that cells express in a cell cycle-coordinate fashion. VM26 interacts primarily with topoisomerase II, an enzyme whose expression reaches the highest levels in the S–$G_2$M phase of the cell cycle (Kimura et al, 1994). Fast-cycling cells have a higher fraction of cells in this phase and, therefore, are more affected by drug cytotoxicity than slow-cycling cells.

CSA at the dosage we used in the patients probably overcomes drug resistance mediated by P-gp, but inhibition of P-gp activity increases the cytotoxic effect of the antineoplastic drug only in MDR cells in which unresponsivity to the antineoplastic treatment is principally caused by an unpaired intracellular drug accumulation. This could be the case in MDR cells with rapid growth kinetics, such as haemopoietic precursor cells, which are virtually chemosensitive to antineoplastic drugs. Conversely, reversal treatment had no effect on MDR cells with longer doubling times (i.e. RCC), since in these cells the growth kinetic characteristics or other drug-resistant mechanisms not related to P-gp expression, are the major impediment to the efficacy of an antineoplastic drug treatment.

In conclusion, this study demonstrates that CSA affects both the pharmacokinetics and the pharmacodynamics of VM26 in patients with RCC. In tumours with slow growth kinetics, such as RCCs, inhibition of P-gp activity is probably necessary but not sufficient to restore the chemosensitivity to the antineoplastic drugs. In these tumours, new modalities of treatment must be adopted to improve clinical results.

### ACKNOWLEDGEMENT

This study was supported in part by CNR Progetto Finalizzato ACRO (grant 95.00546.PF39) and AIRC.

### REFERENCES

Boiocchi M and Toffoli G (1992) Mechanism of multidrug resistance in human tumour cell lines and complete reversion of cellular resistance. *Eur J Cancer* **28A:** 1099–1105

Charuk JH, Wong PY and Reithmeier RA (1995) Differential interaction of human renal P-glycoprotein with various metabolites and analogues of cyclosporin A. *Am J Physiol* **269:** F31–F39

Chaudhary PM and Roninson IB (1991) Expression and activity of P-glycoprotein, a multidrug efflux pump, in human hematopoietic stem cells. *Cell* **66:** 85–94

Clark PI and Slevin ML (1987) The clinical pharmacology of etoposide and teniposide. *Clin Pharmacokinet* **12:** 223–252

Evans WK, Sinkule JA and Crom WR (1982) Pharmacokinetics of teniposide (VM26) and etoposide (VP26-213) in children with cancer. *Cancer Chem Pharmacol* **7:** 147–150

Fojo AT, Ueda K, Slamon DJ, Poplack DG, Gottesman MM and Pastan I (1987) Expression of a multidrug-resistance gene in human tumors and tissues. *Proc Natl Acad Sci USA* **84:** 265–269

Ford JM and Hait WN (1990) Pharmacology of drugs that alter multidrug resistance in cancer. *Pharmacol Rev* **42:** 155–199

Fridborg H, Jonsson B, Nygren P, Csoka K, Nilsson K, Oberg G, Kristensen J, Bergh J, Tholander B, Olsen L, Jakobson A and Larsson R (1994) Activity of cyclosporins as resistance modifiers in primary cultures of human haematological and solid tumours. *Br J Cancer* **70:** 11–17

Gosland MP, Brophy NA, Duran GE, Yahanda AM, Adler KM, Hardy RI, Halsey J and Sikic BI (1991) Bilirubin: a physiological substrate for the multidrug transporter. *Proc Annu Meet Am Assoc Cancer Res* **32:** A2533

Kimura K, Saijo M, UI M and Enomoto T (1994) Growth state- and cell cycle-dependent fluctuation in the expression of two forms of DNA topoisomerase II and possible specific modification of the higher molecular weight form in the M phase. *J Biol Chem* **269:** 1173–1176

Klimecki WT, Futscher BW, Grogan TM and Dalton WS (1994) P-glycoprotein expression and function in circulating blood cells from normal volunteers. *Blood* **83:** 2451–2458

Lehnert M (1993) Reversal of P-glycoprotein-associated multidrug resistance: the challenge continues. *Eur J Cancer* **29A:** 636–638

Lum BL, Kaubisch S, Yahanda AM, Adler KM, Jew L, Ehsan MN, Brophy NA, Halsey J, Gosland MP and Sikic BI (1992) Alteration of etoposide pharmacokinetics and pharmacodynamics by cyclosporine in a phase I trial to modulate multidrug resistance. *J Clin Oncol* **10:** 1635–1642

Maurer G and Lemaire M (1986) Biotransformation and distribution in blood of cyclosporine and its metabolites. *Transplant Proc* **18:** 25–34

Meador J, Sweet P, Stupecky M, Wetzel M, Murray S, Gupta S and Slater L (1987) Enhancement by cyclosporin A of daunorubicin efficacy in Ehrlich ascites carcinoma and murine hepatoma 129. *Cancer Res* **47:** 6216–6219

Osieka R, Seeber S, Pannenbacker R, Soll D, Glatte P and Schmidt CG (1986) Enhancement of etoposide-induced cytotoxicity by cyclosporin A. *Cancer Chemother Pharmacol* **18:** 198–202

© Cancer Research Campaign 1997

Raderer M and Scheithauer W (1993) Clinical trials of agents that reverse multidrug resistance. A literature review. *Cancer* **72:** 3553–3563

Relling MV, Nemec J, Schuetz EG, Schuetz JD, Gonzalez FJ and Korzekwa KR (1994) *O*-demethylation of epipodophyllotoxins is catalyzed by human cytochrome P450 3A4. *Mol Pharmacol* **45:** 352–358

Rodenburg CJ, Nooter K, Herweijer H, Seynaeve C, Oosterom R, Stoter G and Verweij J (1991) Phase II study of combining vinblastine and cyclosporin-A to circumvent multidrug resistance in renal cell cancer. *Ann Oncol* **2:** 305–306

Schuurhuis GJ, Broxterman HJ, Cervantes A, Van Heijningen TH, DE Lange JH, Baak JP, Pinedo HM and Lankelma J (1989) Quantitative determination of factors contributing to doxorubicin resistance in multidrug-resistant cells. *J Natl Cancer Inst* **81:** 1887–1892

Skovsgaard T (1978) Mechanism of cross-resistance between vincristine and daunorubicin in Ehrlich ascites tumor cells. *Cancer Res* **38:** 4722–4727

Sonneveld P and Nooter K (1990) Reversal of drug-resistance by cyclosporin-A in a patient with acute myelocytic leukaemia. *Br J Haematol* **75:** 208–211

Sonneveld P, Durie BG, Lokhorst HM, Marie JP, Solbu G, Suciu S, Zittoun R, Lowenberg B and Nooter K (1992) Modulation of multidrug-resistant multiple myeloma by cyclosporin. The Leukaemia Group of the EORTC and the HOVON. *Lancet* **340:** 255–259

Taylor RJ (1990) *Introduzione all'analisi degli errori*. pp. 202–203. Zanichelli: Bologna

Tew KD, Houghton PJ and Houghton JA (1993) *Preclinical and Clinical Modulation of Anticancer Drugs*. pp. 126. CRC Press: Boca Raton

Toffoli G, Simone F, Corona G, Raschack M, Cappelletto B, Gigante M and Boiocchi M (1995) Structure–activity relationship of verapamil analogs and reversal of multidrug resistance. *Biochem Pharmacol* **50:** 1245–1255

Toffoli G, Corona G, Gigante M and Boiocchi M (1996) Inhibition of P-gp activity and cell cycle dependent chemosensitivity to doxorubicin in multidrug-resistant LoVo human colon cancer cell line. *Eur J Cancer* **32A:** 1591–1597

Twentyman PR (1992) Cyclosporins as drug resistance modifiers. *Biochem Pharmacol* **43:** 109–117

Verweij J, Herweijer H, Oosterom R, Van Der Burg ME, Planting AS, Seynaeve C, Stoter G and Nooter K (1991) A phase II study of epidoxorubicin in colorectal cancer and the use of cyclosporin-A in an attempt to reverse multidrug resistance. *Br J Cancer* **64:** 361–364

Vincent PC (1983) Kinetics of leukemia and control of cell division and replication. In *Leukemia*, Gunz FW and Henderson ES (eds), pp. 77–118. Grune & Stratton: New York

Warner E, Tobe SW, Andrulis IL, Pei Y, Trachtenberg J and Skorecki KL (1995) Phase I–II study of vinblastine and oral cyclosporin A in metastatic renal cell carcinoma. *Am J Clin Oncol* **18:** 251–256

Wilson WH, Jamis Dow C, Bryant G, Balis FM, Klecker RW, Bates SE, Chabner BA, Steinberg SM, Kohler DR and Wittes RE (1995) Phase I and pharmacokinetic study of the multidrug resistance modulator dexverapamil with EPOCH chemotherapy. *J Clin Oncol* **13:** 1985–1994

Yagoda A, Abi Rached B and Petrylak D (1995) Chemotherapy for advanced renal-cell carcinoma: 1983–1993. *Semin Oncol* **22:** 42–60

Yahanda AM, Adler KM, Fisher GA, Brophy NA, Halsey J, Hardy RI, Gosland MP, Lum BL and Sikic BI (1992) Phase I trial of etoposide with cyclosporine as a modulator of multidrug resistance. *J Clin Oncol* **10:** 1624–1634

© Cancer Research Campaign 1997